# Exploring biomarkers and molecular mechanisms of Type 2 diabetes mellitus promotes colorectal cancer progression based on transcriptomics

**DOI:** 10.1038/s41598-025-88520-4

**Published:** 2025-02-03

**Authors:** Simin Luo, Yuhong Zhu, Zhanli Guo, Chuan Zheng, Xi Fu, Fengming You, Xueke Li

**Affiliations:** 1 Traditional Chinese Medicine Regulating Metabolic Diseases Key Laboratory of Sichuan Province, Hospital of Chengdu University of Traditional Chinese Medicine, Chengdu, 610075 Sichuan China; 2 Oncology Teaching and Research Department, Chengdu University of Traditional Chinese Medicine, Chengdu, 610075 Sichuan China; 3 Institute of Oncology, Chengdu University of Traditional Chinese Medicine, Chengdu, 610075 Sichuan China

**Keywords:** Colorectal cancer, Type 2 diabetes, COX11, Disease markers, Pathogenesis

## Abstract

Type 2 diabetes mellitus (T2DM) has been confirmed as an independent risk factor for colorectal cancer (CRC) in many studies. However, the mechanisms behind T2DM’s role in the progression of CRC remain unclear. This study aims to explore the potential biomarkers and molecular mechanisms involved in T2DM-promoted CRC progression. The limma package was used to identify differentially expressed genes in tumor tissue from CRC patients with or without T2DM. The key biological processes were screened by gene ontology and gene set enrichment analysis. A diagnostic model for co-morbidities was constructed by logistic regression model with least absolute shrinkage and selection operator (Lasso) regularization method. The diagnostic performance was assessed by supplementing external datasets to draw ROC curves on the diagnostic model. The diagnostic model was further screened for key genes by prognostic analysis. The relationship of key genes with immune cells and other cells was evaluated by immune infiltration algorithm and single-cell transcription analysis. Drug prediction was performed by cMAP and the obtained drugs were molecularly docked with the key genes. The differentially expressed genes of T2DM-promoted CRC progression were mainly enriched to O-linked glycosylation-related processes. The diagnostic model constructed based on Lasso logistic regression had good diagnostic performance (AUC > 0.8). COX11 was the key gene for co-morbidities: in tumor tissues, COX11 expression was significantly higher than that in normal colon tissues. However, *COX11* gene expression was significantly lower in patients with comorbidities than in patients without T2DM in tumor tissue. External datasets confirmed from both mRNA and protein expression levels that low *COX11* expression was significantly associated with poor CRC prognosis. Immune infiltration analysis suggested that its expression related to the proportion of M2 macrophages. Single-cell transcriptome analysis revealed a close association of *COX11* expression with endothelial cells and macrophages. The top4 drugs predicted bound well to *COX11*. Our study revealed that the pathogenesis of T2DM-promoted CRC progression related to O-linked glycosylation. We constructed a diagnostic model for T2DM-CRC co-morbidity. Meanwhile, we identified *COX11* as a potential immune-related molecular marker closely associated with T2DM-promoted CRC progression. These mechanisms and molecular markers may provide new ideas for further studies of T2DM-promoted CRC progression and contribute to drug discovery for the treatment of co-morbidities.

**Supplementary Information:**

The online version contains supplementary material available at 10.1038/s41598-025-88520-4.

## Introduction

Colorectal cancer (CRC), including colon adenocarcinoma (COAD) and rectum adenocarcinoma (READ), is one of the most common malignancies and a leading cause of cancer deaths worldwide. CRC is susceptible to comorbidities, which can complicate the disease and affect treatment and survival^1^, as well as create a greater economic and social burden. In a retrospective cohort study of 29,733 CRC patients, nearly 4% of deaths were attributed to diabetes^2^. This simultaneous presence of two or more chronic diseases in the same individual is known as co-morbidity^3^. Diseases are often not randomly clustered, and co-morbidities may have common underlying risk factors behind them; or complications or treatments for some diseases lead to the development of another. Exploring disease associations can enhance understanding of co-morbidities, which can further improve disease diagnosis, prognosis, and therapeutic approaches^4^. Therefore, it is essential to study the molecular mechanisms of co-morbidities.

A number of observational studies have reported an association between type 2 diabetes and CRC risk. In 2006, Paul J Limburg et al. conducted a cohort study^5^ and found that T2DM was associated with an increased risk of CRC. A multicenter cohort in Korea^6^ also observed that high fasting glucose and a history of diabetes were linked to increased risk of CRC. In addition to increasing the risk of CRC, diabetes mellitus can affect the prognosis of CRC patients^7^. In CRC patients with T2DM, unhealthy glucose management is linked to a clinically severe cancer course^8^. Although most of this evidence comes from epidemiologic studies, which may have limitations due to diagnostic bias and confounding factors, it does suggest an association between T2DM and CRC to some extent. Meanwhile, metformin, a glucose-lowering drug clinically used to treat T2DM, has demonstrated efficacy in lowering the incidence of CRC^9–11^. In 2021, the American Gastroenterological Association (AGA), in a clinical practice update on chemoprevention of colorectal neoplasms^12^, suggested that: for patients with T2DM, clinicians may think about preventing CRC by using metformin. For patients with co-morbidities of CRC and T2DM, clinicians might think about utilizing metformin to lower mortality. Further basic research has also found that metformin may increase survival in patients with T2DM-CRC co-morbidities by suppressing the epithelial mesenchymalization phenotype^13^, the immune microenvironment^14^, and the gut microbiota^15^. This evidence further supports the association of T2DM-promoting CRC progression.

Some scholars have explored the mechanism of T2DM promotes CRC progression from genetic variation of T2DM susceptibility genes^16,17^, IGF-1 levels^18–21^, and gut microbes^22,23^. Currently, there are fewer studies exploring molecular mechanisms from the level of the co-morbid transcriptome. Although some bioinformatics studies on T2DM-CRC comorbidities^24–27^ have been reported, most of the samples they used were samples with T2DM or CRC alone, rather than samples with co-morbidities. In fact, samples from patients with CRC who have T2DM are more plausible in presenting a co-morbid gene expression profile. We therefore designed, and conducted, the present study to explore the molecular mechanisms by which T2DM promotes CRC progression.

**Fig. 1:**
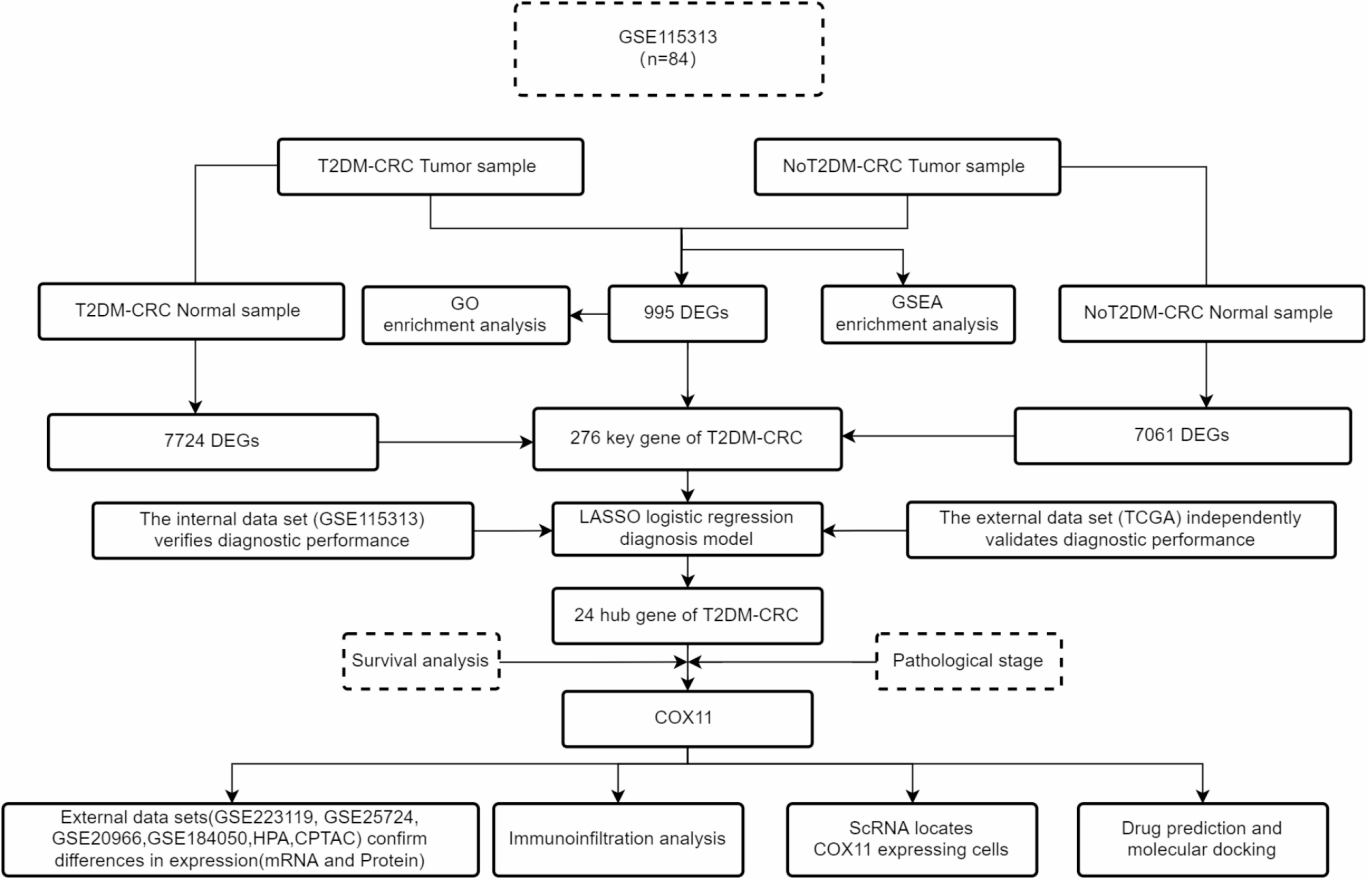
Workflow diagram for exploring the molecular mechanisms by which T2DM promotes CRC progression. (T2DM-CRC: T2DM and CRC comorbidity patients; NoT2DM-CRC: CRC patients without T2DM).

In this study, we focused on key genes linked to T2DM-promoted CRC progression. We analyzed a high-throughput data sequencing dataset (GSE115313) downloaded from the GEO database. An integrated bioinformatics and systems biology approach was used to identify the common differentially expressed genes and their functions in tumor tissues in patients with T2DM-combined CRC and CRC patients without T2DM, and to explore the unique phenotype of T2DM-promoted CRC progression. In addition, sequencing data from patients’ tumor tissues and normal tissues were combined to screen gene clusters and construct diagnostic models by Lasso logistic regression. The performance of the diagnostic models was confirmed using an external dataset (TCGA-COREAD). Key genes in the model were screened by prognostic analysis and evaluated for correlation with pathological staging and immunization. Cells with key gene *COX11* expression were localized by analyzing colon cancer single-cell transcriptome data (GSE231559). Finally, drug prediction and molecular docking were performed. The flow of this study is shown in Fig. 1.

## Materials and methods

### Download of datasets

The main datasets used in this study were as follows: 84 samples were collected in GSE115313, including tumor and normal colon tissues from T2DM-CRC and NoT2DM-CRC patients. 10× single-cell sequencing data from tumor and normal colon tissues from CRC patients were collected in GSE231559. The above datasets were downloaded from the GEO database (www.ncbi.nlm.nih.gov/geo). The dataset for TCGA-COREAD was acquired from UCSC Xena. Other datasets were downloaded from CPTAC (https://gdc.cancer.gov/about-gdc/contributed-genomic-data-cancer-research/clinical-proteomic-tumor-analysis-consortium-cptac), HPA (https://www.proteinatlas.org/) and TISIDB (http://cis.hku.hk/TISIDB/).

### Obtaining T2DM-CRC DEGs

The limma package was utilized to perform differential analysis, and identify the differentially expressed genes (DEGs) in tumor tissues of patients with T2DM-CRC and NoT2DM-CRC. The genes with *p* < 0.05 were taken as differentially expressed genes, in which log2FoldChange > 0 was up-regulated differentially expressed genes and log2FoldChange < 0 was down-regulated differentially expressed genes. The obtained groups of differentially expressed genes were plotted as venn plots to take the intersection. The downstream differentially expressed gene clusters of T2DM promoting CRC development were identified. Volcano plots were drawn to show the results of differential analysis.

### Enrichment analysis

DEGs were obtained and performed Gene ontology enrichment analysis using DAVID^28^ and metaScape. These results were visualized by drawing bubble plots. Using GSEA 4.3.3 software, Gene Set Enrichment Analysis (GSEA) was carried out on the gene expression matrix. These *p* < 0.05 enrichment results were considered statistically significant.

### Least absolute shrinkage and selection operator regression to construct a diagnostic model

Least absolute shrinkage and selection operator (Lasso) regression is a machine learning algorithm that has been widely used to screen signature genes and construct models for various diseases, such as gastrointestinal diseases^29^. We construct a logistic regression model (lasso-logistic regression) with Lasso regularization. The analysis was performed using the glmnet package, setting the number of random seeds, and after inputting the gene expression matrix, setting up ten-fold cross-validation to train the model.

### Plotting ROC curves

Receiver Operating Characteristic (ROC) curves are most typically used in medicine as a technique of assessing diagnostic tests^30^. The TCGA-COREAD and internal datasets were analyzed using the pROC package, and ROC curves were generated to evaluate the model’s diagnostic performance.

### Prognostic analysis and validation

Using GEPIA 2^31^ for survival analysis of each gene in the diagnostic model. In the meantime, an analysis was conducted on the gene expression levels of various disease stages. The correlation between the genes in the diagnostic model and CRC prognosis was assessed. After screening to obtain genes significantly correlated with CRC prognosis, the expression levels (mRNA and protein relative expression levels) and prognostic significance of the key genes were again verified using the GSE223119, GSE25724, GSE20966, GSE184050 datasets, HPA database, and CPTAC database.

### Immune correlation analysis

Cibersoft^32^ is an algorithm for analyzing the proportion of immune cells that has been widely used to assess the level of immune infiltration in oncologic and non-oncologic diseases. Twenty-two immune cell ratios from sequencing data were assessed using Cibersoft. Correlations between key gene expression levels and immune cell ratios were calculated and correlation heatmaps were drawn. Evaluate the correlation between key genes and immunity based on the TISIDB database.

### Single-cell transcriptome analysis

10 × single-cell transcriptome data were extracted using the Seurat package, and the data were normalized after removing mitochondrial genes with expression greater than 20% and genes with expression less than 200. After PCA downscaling, the appropriate number of Clusters were filtered by JackStraw and ElbowPlot methods to draw umap sub-cluster plots. Individual Clusters were annotated using singleR package with celldex package. The annotated cell clusters were differentiated into normal and tumor tissues, and the cells expressing key genes were located at the same time.

### Drug prediction & molecular docking

DEGs from tumor samples of patients with T2DM-CRC co-morbidities versus CRC-only patients were uploaded to cMAP^33^, and the reference cell line was HT29 (colon cancer cell line) for drug prediction. Structure files of predicted drugs were obtained from Pubchem. Structure files of key genes were downloaded from AlphaFoldDB database (https://alphafold.ebi.ac.uk/). Molecular docking was performed using CB-DOCK2^34^ to assess the level of drug binding to key genes.

## Results

### T2DM-CRC differentially expressed genes analysis

**Fig. 2:**
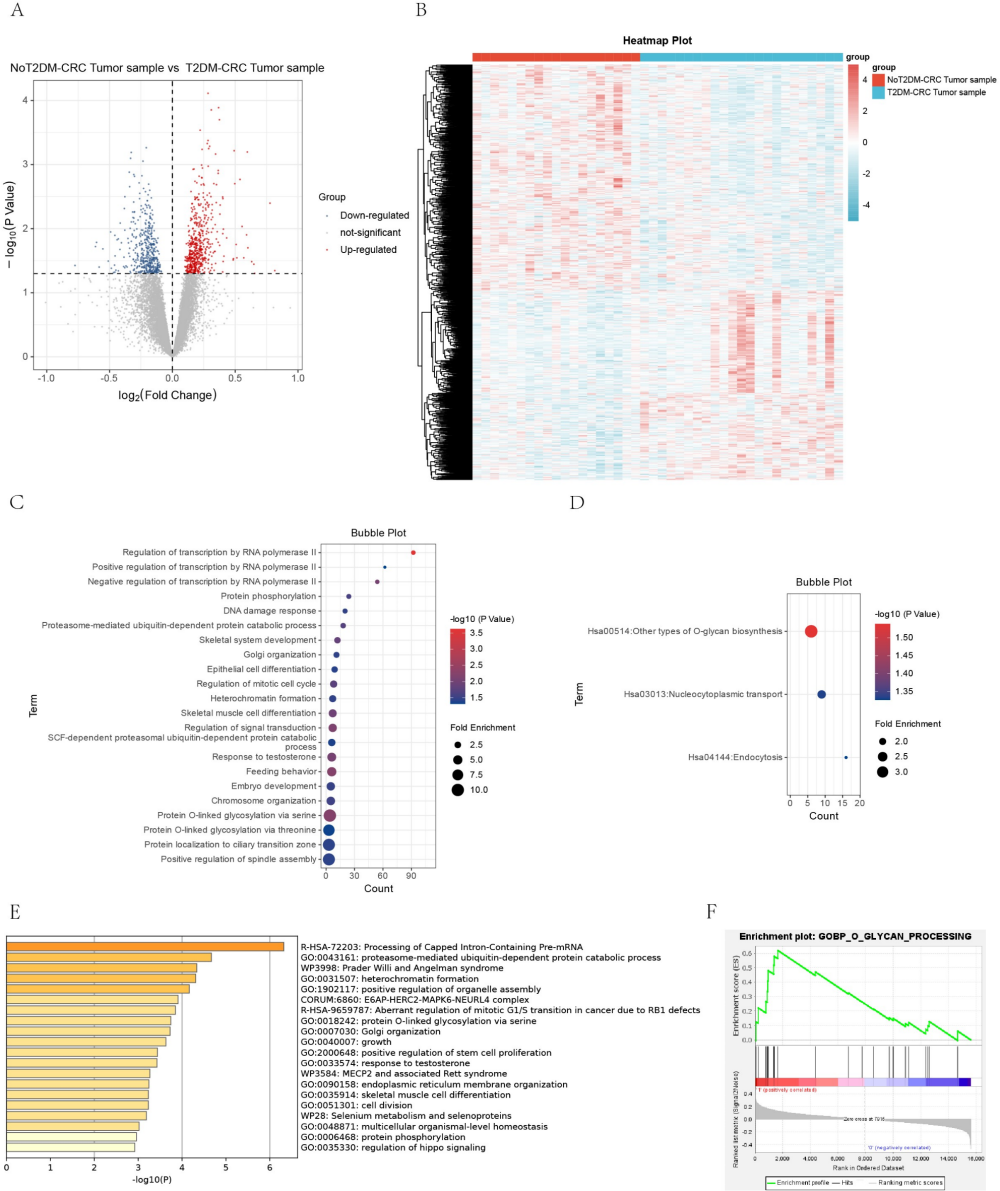
T2DM promotes CRC progression and is strongly associated with O-linked glycosylation. (**A**) Volcano plot of differential gene analysis of tumor tissues from CRC patients without T2DM versus CRC patients with T2DM. (**B**) Heatmap of expression clustering of differentially expressed genes. (**C**,**D**) O-linked glycosylation-associated processes were significantly enriched by GO-BP versus KEGG^35–37^ enrichment analysis (DAVID). (**E**) Go BP enrichment analysis (METESCAPE). (**F**) O-glycan-related processes were significantly upregulated in T2DM-CRC co-morbidities (GSEA).

A total of 995 DEGs were obtained by differential analysis (Fig. 2A). The expression levels of these differentially expressed genes between the two groups were displayed using the clustering heat map (Fig. 2B).

### T2DM promotes CRC progression associated with O-linked glycosylation

The 995 DEGs were subjected to enrichment analysis by the DAVID database. These DEGs are mainly associated with O-linked glycosylation biological processes, such as protein O-linked glycosylation via serine or via threonine (Fig. 2C). Meanwhile, from the results of KEGG enrichment, we observed that these DEGs are mainly involved in O-glycan biosynthesis, endocytosis and other pathways (Fig. 2D). Considering the limitations of a single database, enrichment analysis was supplemented using the metascape database. Enrichment of these differential genes to O-linked glycosylation biological processes was similarly observed (Fig. 2E). The gene expression data were uploaded to GSEA, which revealed a significant upregulation of O-linked glycosylation-related biological processes (Fig. 2F).

### Diagnostic model construction and validation

**Fig. 3:**
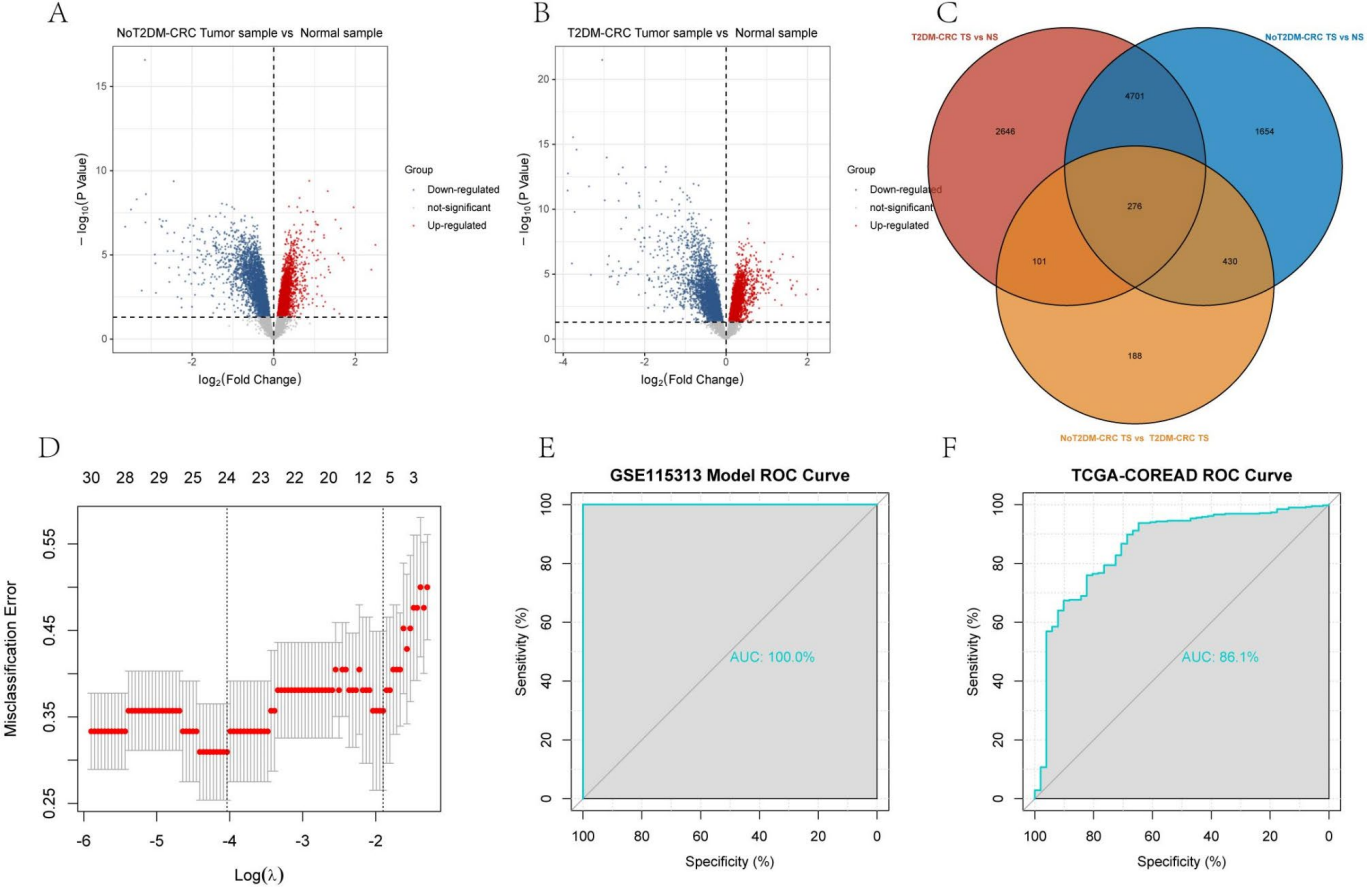
Constructing diagnostic models based on Lasso logistic regression. (**A**–**C**) Tumor tissues and normal tissues of CRC patients were analyzed for differentially expressed genes, and the intersection set was taken to screen the set of differentially expressed genes for T2DM-CRC co-morbidities. (**D**) Lasso logistic regression was used to screen the key variables and construct the diagnostic models. (**E**) ROC curves were plotted to assess the diagnostic performance using GSE115313 as an internal dataset. (**F**) ROC curves were plotted to assess the diagnostic performance using TCGA-COREAD as an external validation dataset.

Tumor samples and healthy samples of CRC patients without T2DM were differentially analyzed, and tumor samples and healthy samples of CRC patients with combined T2DM were differentially analyzed. The differentially expressed genes of combined T2DM combined CRC were taken as intersection, and a total of 276 genes were obtained (Fig. 3A–C). These genes were closely associated with T2DM promoting CRC development.

Diagnostic models were constructed for these genes using Lasso logistic regression. After setting the number of random seeds and ten-fold cross-validation, diagnostic models constructed for 24 genes were obtained (Fig. 3D). The diagnostic performance was validated using internal dataset, and its model had an excellent performance. The external dataset validation supplementing TCGA also had a good performance (AUC > 0.8) (Fig. 3E,F).

### Screening *COX11* as a key gene in T2DM for CRC progression

**Fig. 4:**
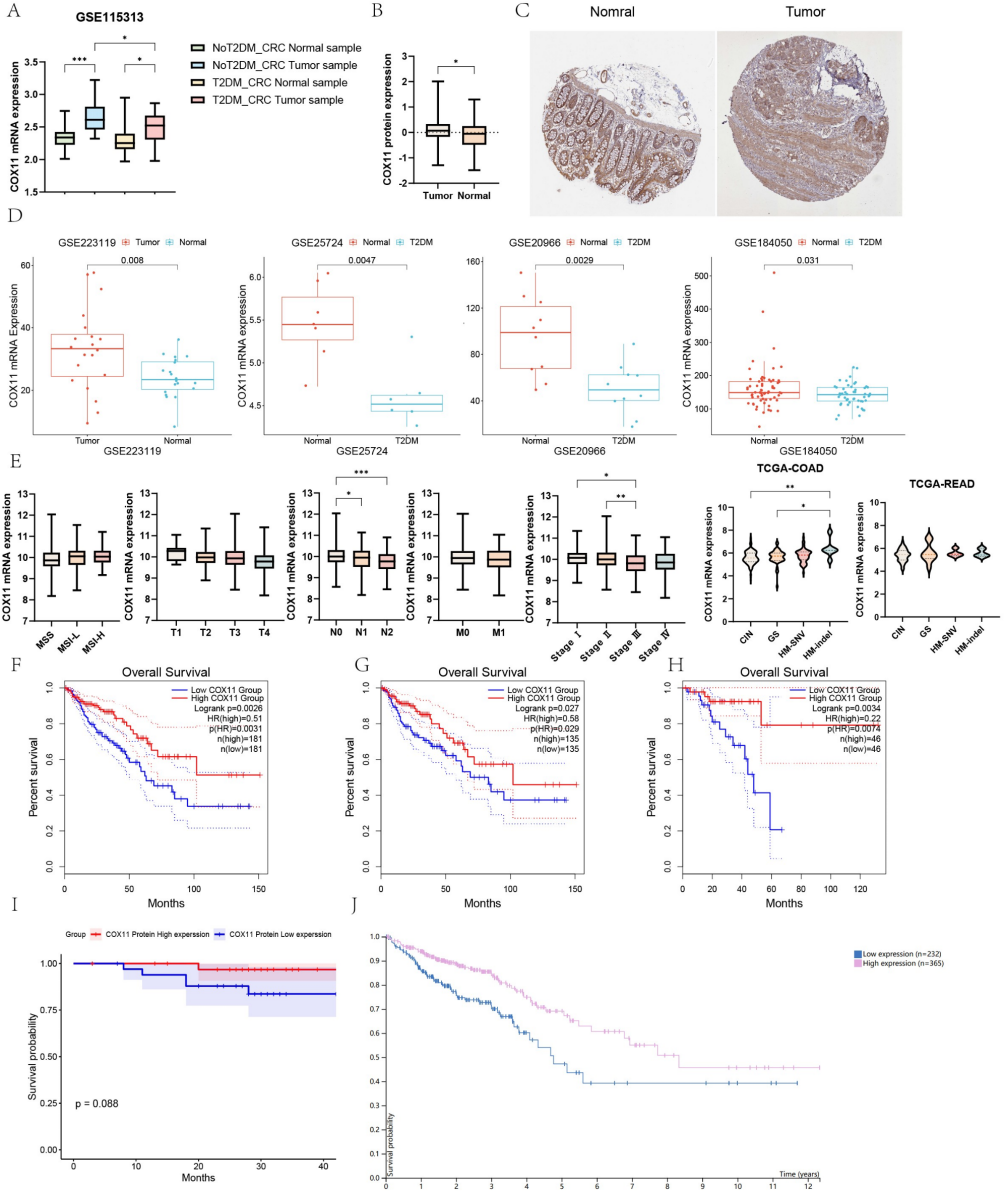
Low expression of *COX11* in tumor tissues of patients with T2DM-CRC co-morbidities was significantly associated with poor prognosis. (**A**) Expression of *COX11* in tumor tissues and normal tissues of patients with CRC. (**B**,**C**). Proteomic data and HPA databases confirmed that the relative expression level of COX11 protein is significantly up-regulated in CRC tumor tissues. (**D**) External datasets confirmed at the transcriptional level that *COX11* is highly expressed in tumor tissues and low expressed in T2DM pancreatic islets. (**E**) *COX11* was significantly correlated with pathological stage and N stage, and not significantly correlated with MSI stability and T and M stage. tissues and low expression in T2DM islets. (**F**–**H**). Low expression of *COX11* (at transcriptional level) in TCGA-COREAD(F)/TCGA-COAD(G)/TCGA-READ(H) is suggestive of poor prognosis. (**I**) COX11 survival curves based on CPTAC data (protein level). (**J**) *COX11* survival curves obtained based on HPA (transcript level) database analysis.

Analysis of the survival prognosis and pathological correlations of 24 genes in the diagnostic model by GEPIA 2 revealed that only *COX11* was associated with prognosis and pathological staging, and the expression trend was consistent with that in T2DM combined with CRC (Supplementary Figs. 1–2). In contrast to normal tissues, the mRNA expression level of *COX11* was significantly increased with or without combined T2DM (Fig. 4A). In contrast, in tumor tissues, a significant decrease in *COX11* expression was observed in samples with combined T2DM. In the proteomic dataset, COX11 protein expression levels were found to be significantly higher in tumor samples from CRC patients. This effect was also observed on immunohistochemical staining (Fig. 4B,C). In CRC tumor tissues, there was an increase in the mRNA expression level of *COX11* in the external dataset, whereas in T2DM patients, there was a notable drop in the mRNA expression level of *COX11* (Fig. 4D).

### *COX11* is strongly associated with CRC prognosis

Extraction and analysis of the data in TCGA suggested that *COX11* mRNA expression levels were not significantly correlated with microsatellite instability (MSI) and T and M staging (Fig. 4E). *COX11* mRNA expression levels were significantly correlated with pathological staging and N staging. The highest *COX11* expression level was observed in COAD in HM-indel, whereas this correlation was not observed in READ. In the combined COAD-READ dataset (Fig. 4F), COAD (Fig. 4G) dataset, READ (Fig. 4H) dataset, increased *COX11* mRNA expression levels were observed as a feature of good prognosis. Protein expression levels and prognosis, extracted and analyzed from the CPTAC dataset (CPTAC-2 Prospective, Cell 2019)^38^ of protein expression data for prognostic analysis (Fig. 4I). We similarly observed that low COX11 expression levels were associated with poor prognosis, not statistically significant (*p* = 0.088). The HPA database similarly suggested that *COX11* is a good prognostic biomarker for CRC (Fig. 4J).

### Analysis of *COX11* and immune correlations

**Fig. 5:**
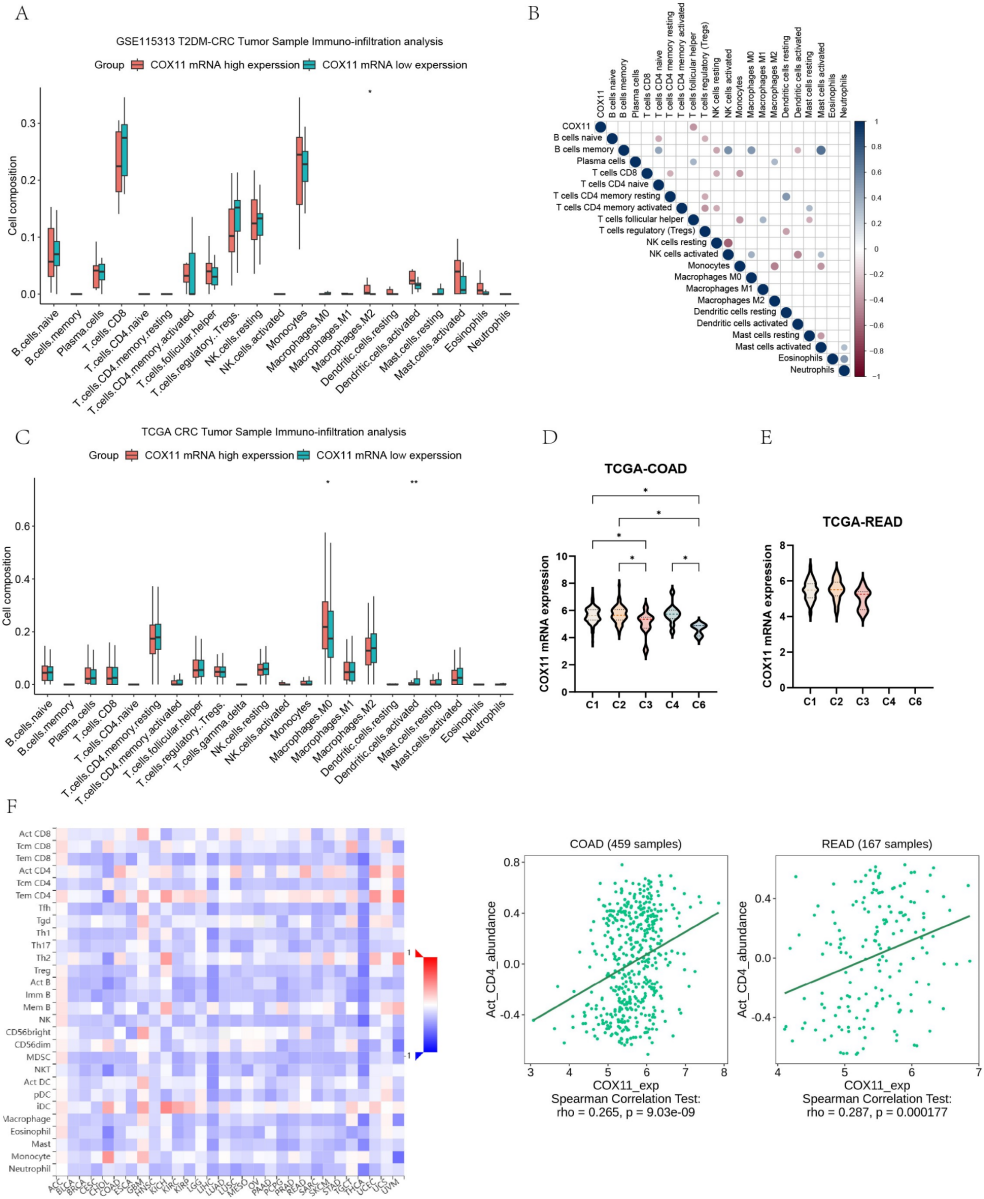
*COX11* is one of immune correlation gene in T2DM-CRC. (**A**) Proportion of immune cells in tumor tissues of patients with co-morbidities in the *COX11* low-expression versus high-expression group. (**B**) Correlation between *COX11* expression level and immune cells in tumor tissues of patients with co-morbidities. (**C**) Proportion of immune cells in tumor samples of patients with TCGA-COREAD in the *COX11* low-expression versus high-expression group. (**D**,**E**) In COAD, READ, the Correlation of *COX11* expression level with immune subtypes. (**F**) A heat map of immune cells and tumor diseases constructed based on MISIDB revealed that the percentage of CD4 T cells and the amount of *COX11* expression in COAD, READ, were favorably associated.

In tumor tissues from patients with combined T2DM-CRC, there was a notable rise in the percentage of M2 macrophages in the *COX11* high-expression group. But correlation thermograms did not observe a correlation between *COX11* expression levels and M2 macrophage proportions (Fig. 5A,B). In tumor tissues from TCGA-COREAD patients, the *COX11* high-expression group showed a significant drop in activated dendritic cells and a large rise in the number of M0 macrophages (Fig. 5C). Significantly lower expression of *COX11* than the other immune subtypes was observed in COAD in C3 (inflammation), and C6 (TGF-β dominant), which was not observed in READ (Fig. 5D,E). In *COX11* expression correlated with CD4 T cells in a certain positive proportion (Fig. 5F).

### Single-cell transcriptome analysis

**Fig. 6:**
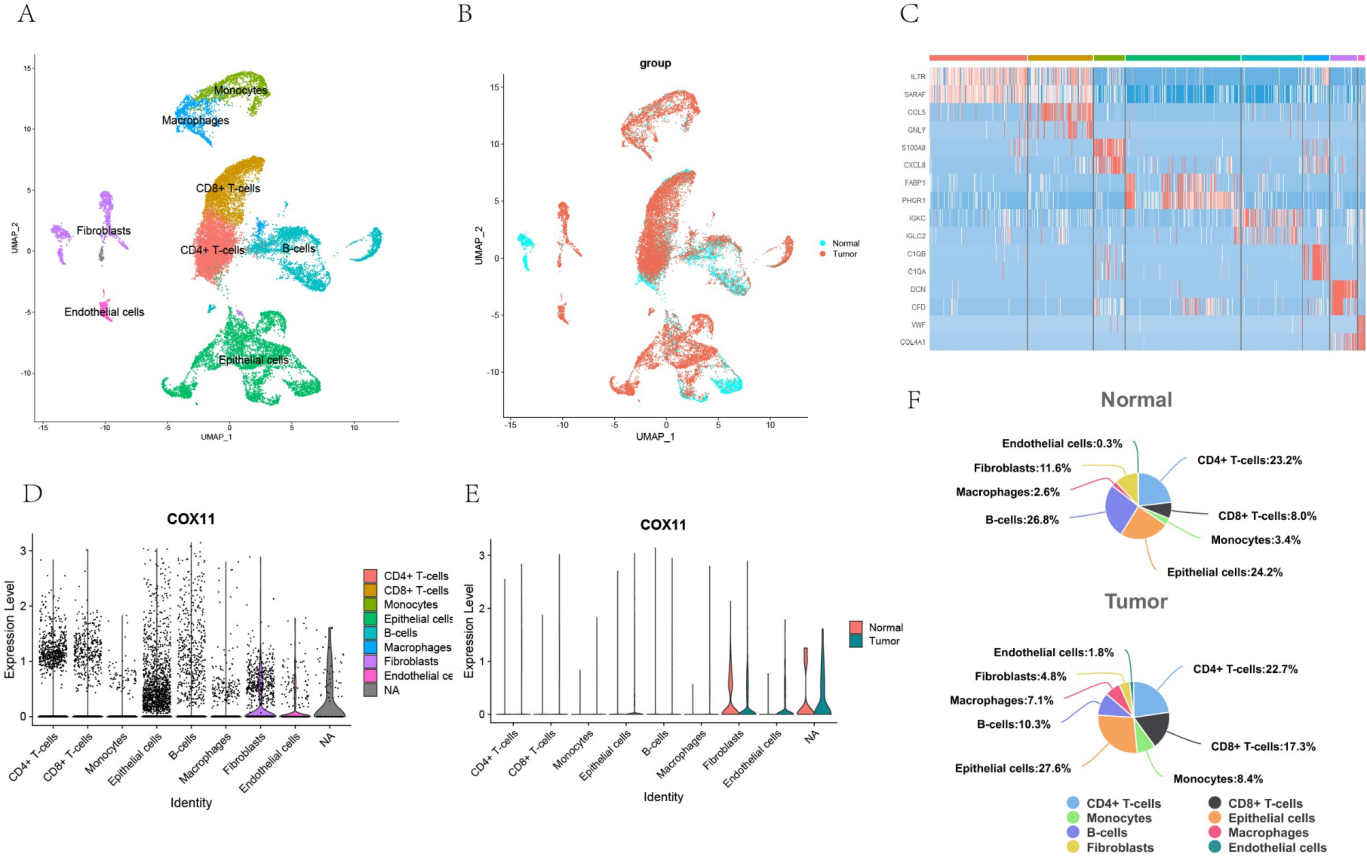
Single-cell transcriptome validation of *COX11* expression in tumor versus normal colon tissues of CRC patients. (**A**,**B**) Cellular clustering of tumor tissues versus normal colon tissues. (**C**) Heatmap of marker genes in cellular clusters. (**D**,**E**) Expression of *COX11* in tumor tissues versus normal colon tissues of different cells. (**F**) Percentage of different cells in tumor tissues versus normal colon tissues.

After annotation, tumor tissues were mainly divided into 8 cell clusters with normal colon tissues (Fig. 6A,B). Among them, Fig. 6C demonstrated part of the marker that distinguished the cell clusters. In tumor tissues, a trend of increasing *COX11* expression levels was observed in cells such as endothelial cells and macrophages (Fig. 6D,E). There was an increase in the proportion of CD8 + T lymphocytes to epithelial cells in tumor tissues (Fig. 6F).

### COX11 binds well to predictive drugs

**Fig. 7:**
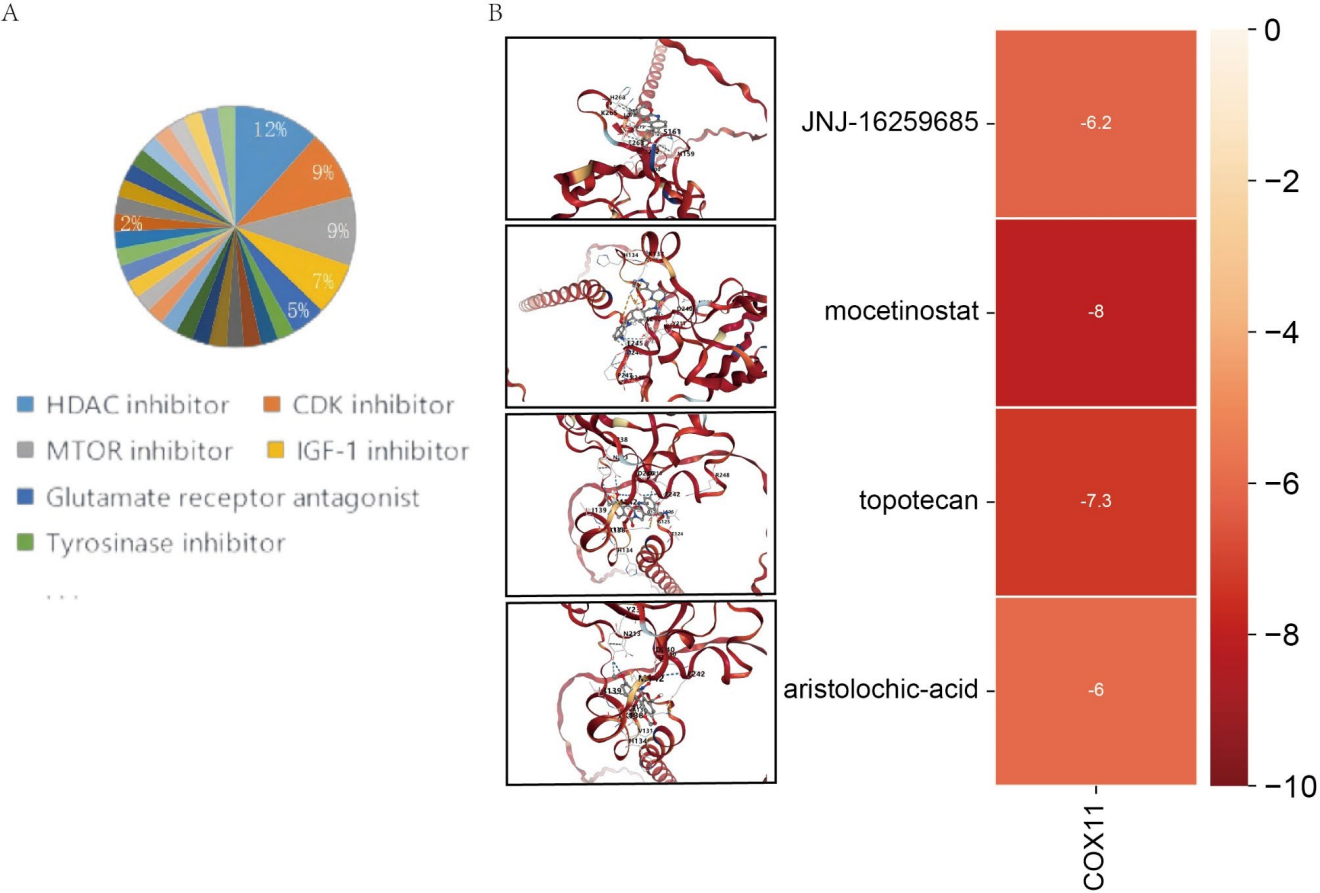
Molecular docking plots of drugs with *COX11* based on cMAP prediction. (**A**) Categorical percentage of cMAP-predicted drugs (prediction scores < -90). (**B**) Docking binding sites are shown on the left, the names of drugs binding to *COX11* are shown in the middle region, and the heatmap of the binding scores of each drug binding to *COX11* is shown on the right (this diagram was drawn by author: *Luo Simin*).

Tumor tissue sequencing matrix differentially expressed genes from co-morbid versus CRC-only patients were uploaded to the cMAP database, with the reference cell line HT29 (colon cancer cell line). HDAC inhibitors were found to have the highest percentage (12%) of drugs with a predicted score of < -90 (Fig. 7A). The top 4 drugs with negative scores obtained were JNJ-16259685, topotecan, mocetinostat, and aristolochic-acid. The molecular docking results (Fig. 7B) suggested that they could dock well in Table 1 (Vina score < -4).

**Table 1:** Molecular docking with *COX11* based on cMAP-predicted high relevance score drug information (TOP4).

Name	Score	ID	Description	Vina score	Docking size
JNJ-16259685	-99.27	BRD-K64670467	Glutamate receptor antagonist	-6.2	23, 23, 23
Topotecan	-99.22	BRD-A59985574	Topoisomerase inhibitor	-7.3	24, 24, 24
Mocetinostat	-98.72	BRD-K16485616	HDAC inhibitor	-8	27, 27, 27
Aristolochic-acid	-98.62	BRD-K17110974	Phospholipase inhibitor	-6	20, 20, 20

## Discussion

CRC is currently the second leading cause of cancer deaths worldwide. With the accumulation of clinical data, a large number of studies have reported the association between T2DM and CRC risk. T2DM has received increasing attention as an independent risk factor for CRC. One cohort study^39^ found that patients with T2DM had a significantly higher risk of cancer death than non-diabetic patients. Another cohort study^40^ that included 59,202 patients with CRC found that patients with colorectal cancer combined with diabetes also had a higher risk of recurrence than patients without diabetes. Metformin has been reported to significantly improve overall survival^41^ and disease-free survival^42^ in patients with T2DM-CRC co-morbidities. Currently, some studies have pointed out that pathological processes inherent to T2DM such as chronic hyperglycemia, insulin resistance, oxidative stress, and inflammation also play an active role in colorectal carcinogenesis and progression^43^. These may be the molecular mechanisms by which T2DM promotes CRC progression. Some studies^24–27^ have also explored the related molecular mechanisms from a transcriptomic perspective. Some of these studies integrated large samples with single-cell transcriptomes and eliminated the batch effect, which has some credibility. However, the sequencing data used were not from co-morbid patient tissues, but samples from single T2DM or CRC patients. It is therefore difficult to accurately characterize the gene expression profile of tumor tissues in the T2DM-CRC co-morbid state. In order to explore the molecular mechanisms of T2DM-promoted CRC progression more comprehensively, we designed the present study.

First, we performed differential expression analysis and enrichment analysis on the normalized sequencing data. The DEGs obtained were mainly enriched for related processes such as O-linked glycosylation (a type of glycosylation). Glycosylation is a way in which proteins and lipids are modified with complex carbohydrates called glycans^44^. Glycans regulate tumor proliferation, invasion, metastasis, and angiogenesis, among other aspects of tumor growth. Because of this, aberrant glycosylation is a prime contender for cancer diagnostics^45^. MC38 colon cancer cells cultured under high glucose showed more abundant N-glycans^46^. These evidences suggest a close association of glycosylation with CRC. Other studies provide further possible directions for the link between O-linked glycosylation and T2DM-promoted CRC progression. For example, O-GlcNAcylation, a post-translational covalent modification of β-N-acetylglucosamine (O-GlcNAc) molecules on protein serine or threonine residues via O-linkage, has been closely associated with both diabetes and cancer^47^. Upregulation of fatty acid synthase expression increases O-GlcNAc protein glycosylation and promotes colorectal cancer growth^48^. O-GlcNAcylation negatively regulated by microRNA-101 may promote colorectal cancer metastasis by enhancing the stability and function of EZH2 protein^49^. Moreover, O-GlcNAcylation destabilizes the active tetramer PKM2, which promotes the Warburg effect and bridges metabolic reprogramming in tumor diseases^50^. The chemotherapeutic medication 5-fluorouracil (5-FU) impacts O-GlcNAcylation in non-cancerous and malignant colon cells in vitro by reducing O-GlcNAc transferase production. Moreover, Thiamet-G, an O-GlcNAcase inhibitor, and 5-FU together have a synergistic inhibitory effect on tumor development^51^. The above evidence suggests that upstream regulators can promote CRC progression/metastasis by enhancing O-linked glycosylation. Interestingly, we found that T2DM-CRC patients exhibited stronger levels of O-linked glycosylation. It is then likely that T2DM promotes CRC progression by enhancing O-linked glycosylation. In fact, O-linked glycosylation is indeed strongly associated with T2DM^52,53^. Because the subunits necessary for protein glycosylation are supplied by glucose. Therefore, it is likely that hyperglycemia induce O-linked glycosylation of certain proteins (e.g., O-GlcNAcylation) in T2DM patients, which in turn promotes the development of CRC and triggers a poor prognosis.

Subsequently, we constructed a diagnostic model by Lasso logistic regression, and the diagnostic performance was validated by an external dataset. In order to further explore potential targets closely related to prognosis, we performed prognostic analyses of individual genes in the model, and finally identified *COX11* as a key molecule. We discovered that *COX11* expression levels, both in mRNA and protein, were slightly elevated in tumor tissues. This elevation may be protective in nature. Therefore, when *COX11* expression levels were elevated in tumor tissues, we observed a better prognostic outcome in CRC patients. When the prognostic significance was verified using proteomic data, a trend towards a better prognosis with high expression was observed, but no statistical significance was observed (*p* = 0.088). The reason for this may be that the duration of observation was only 40 months, which is much lower than the duration of observation in databases such as TCGA (150 months). Other studies^54^ have also found that high levels of *COX11* in CRC tumor tissues have a better prognosis. In our validation using an external dataset, we found a significant decrease in *COX11* levels in T2DM patients. Related literature also reported that levels of oxidative phosphorylation genes (e.g., COX11) were significantly decreased in T2DM patients with elevated blood glucose^55^. This implies that low levels of *COX11* brought about by T2DM in patients with concomitant CRC may be a key factor triggering the poor prognosis of CRC. *COX11* is a copper-transporting protein essential for respiratory growth and is associated with the assembly of the Cu(B) site of cytochrome c oxidase^56^. Cellular copper levels are closely related to mitochondrial oxidative phosphorylation, and *COX11* has been reported to interact with several proteins of the same family involved in copper ion transport and oxidative phosphorylation. For example, *COX17*, which transports copper from the cytoplasm to MT-CO1/*COX1* via *COX11*^57,58^. Deletion of *COX 19* of the same family may block the *COX11* response cycle to a phase that is particularly sensitive to redox, leading to over-oxidation and inactivation of *COX11*^59^. These evidences provide a possibility to explain the molecular mechanism by which *COX11* is involved in the progression of co-morbidities. We also explored the relationship of *COX11* with pathological staging and other subtypes. Although *COX11* was associated with pathological staging, its correlation with N staging was only observed. This suggests that *COX11* is significantly associated with high-grade pathological staging and the main reason behind this may be lymph node involvement.

After clarifying that *COX11* is potentially a key molecule in T2DM-promoted CRC progression, we further explored its association with immunity. In tumor tissues of T2DM-CRC patients, the proportion of M2 macrophages was significantly higher in the *COX11* high-expression group. But this effect was not observed in the TCGA-COREAD cohort. The reason for the non-observation may be the lack of T2DM information in the TCGA-COREAD cohort, which does not allow stratification of the T2DM-CRC cohort. We performed a single-cell transcriptome analysis, although the samples were not from co-morbidities but from a single CRC patient. However, it was able to demonstrate to some extent the localization of *COX11* and the differences in its expression levels among different cells. We noticed that an increase in the ratio of endothelial cells to CD8 + T cells in tumor tissues, which may suggest that immunological infiltration of CD8 + T cells took place in tumor tissues. Since *COX11* is localized to mitochondria, we found that *COX11* was somewhat expressed on various cell clusters. In line with the expected trend, in the tumor tissues, we observed an increase in *COX11* expression levels in cells such as endothelial cells and macrophages (although no statistical difference was observed). Combined with the results of immune correlation analysis, we suggest that macrophages may also be key cells for the role of *COX11* in T2DM-promoted CRC progression.

Finally, we performed drug prediction and found that the drugs with high prediction scores were mainly HDAC inhibitors, which mainly act by inhibiting histone deacetylation. While no study has yet demonstrated that HDAC inhibitors can delay the progression of CRC by influencing T2DM, numerous studies have confirmed their efficacy in delaying the progression of either T2DM or CRC. Initially, HDAC inhibitors were incorporated into preclinical and clinical evaluations for gastrointestinal tumors as promising novel therapeutics as early as 2005^60^. Since then, they have been extensively reported to exert antitumor effects, particularly in combination with 5-fluorouracil (5-Fu)^61,62^. Recent research has further elucidated that HDAC inhibitors can impede colon cancer progression or metastasis by modulating various cellular processes, including mitosis^63^, adhesion^64^, apoptosis, epithelial-mesenchymal transition^65^, and drug resistance^66^ in mesenchymal tumor cells. In a separate line of inquiry, a 2014 study identified an association between single nucleotide variants in the HDAC gene and the incidence of T2DM within the Chinese population^67^. Subsequent findings revealed that HDAC inhibitors could enhance β-cell differentiation, proliferation, and function, as well as ameliorate insulin resistance^68,69^. Additionally, HDAC inhibitors have been shown to delay the progression of T2DM by mitigating endoplasmic reticulum stress^70^. Furthermore, HDAC inhibitors have been shown to impede the progression of diabetic complications. For instance, in the context of diabetic cardiomyopathy, HDAC inhibitors have been observed to modulate cardiac PPARs, fatty acid metabolism, and pro-inflammatory cytokines^71^. These findings suggest the potential involvement of the HDAC in the promotion of CRC progression by T2DM. Notably, insulin resistance is a fundamental mechanism underlying metabolic alterations and is believed to be associated with CRC prognosis^72^. Given that HDAC inhibitors have the capacity to ameliorate insulin resistance, we propose that these inhibitors may mitigate the progression of T2DM-CRC co-morbidity by enhancing insulin sensitivity. Future research should focus on evaluating the efficacy and safety of HDAC inhibitors in patients with T2DM-CRC co-morbidities, as well as elucidating the specific mechanisms by which the HDAC contributes to CRC progression in the context of T2DM. Molecular docking suggests that all four drugs predicted by our screening can bind well to the key gene *COX11*, which to some extent demonstrates the potential of *COX11* as a therapeutic target.

In this study, we identified the differentially expressed genes, key genes, and predicted the related molecular mechanisms of T2DM-promoted CRC progression, which helps to further explore the pathogenesis of T2DM-promoted CRC progression. However, our study has some limitations. First, although we used the co-morbidity dataset for our analyses the sample size of this study was only 84 cases, which is on the low side and may affect the credibility of the results. More large samples are needed for validation in the future. Second, although we used an external RNA-seq dataset with a proteomic dataset for validation, we lacked sufficient clinical co-morbidity samples to confirm our findings. Finally, in the future, we will further refine our ex vivo and in vivo experiments to deeply investigate the role *COX11* plays in T2DM-promoted CRC progression.

## Conclusions

In summary, our study revealed that the pathogenesis of T2DM-promoted CRC progression may be related to O-linked glycosylation, and constructed a diagnostic model of T2DM-CRC co-morbidity with certain diagnostic performance. Meanwhile, we identified *COX11* as a potential immune-related molecular marker closely related to T2DM-promoted CRC progression, and found that it might be closely related to macrophages in tumor tissues by immune infiltration and single-cell transcriptome analysis. Overall, these mechanisms and molecular markers may provide new ideas for further mechanistic studies and contribute to drug discovery for its treatment.

## Electronic supplementary material

Below is the link to the electronic supplementary material.

Supplementary Information 1.

Supplementary Information 2.

Supplementary Information 3.

## Data Availability

All datasets analyzed in this study are housed in public databases. The RNA-seq data were deposited into the Gene Expression Omnibus (GEO) database under accession number GSE115313, GSE231559, GSE25724, GSE20966, GSE184050 and are available at the following URL: https://www.ncbi.nlm.nih.gov/geo. The TCGA RNA-seq data were deposited into the UCSC Xena database under accession number TCGA.COADREAD.sampleMap/HiSeqV2 and are available at the following URL: https://xena.ucsc.edu. The protein datasets analysed during the current study were deposited into the cBioPortal for Cancer Genomics under accession number Colon Cancer (CPTAC-2 Prospective, Cell 2019) and are available at the following URL: https://www.cbioportal.org/study/summary?id=coad_cptac_2019.
