# Supplementary figures and images for: Exploring biomarkers and molecular mechanisms of Type 2 diabetes mellitus promotes colorectal cancer progression based on transcriptomics
Source: Sci Rep. 2025 Feb 3;15:4086. doi: 10.1038/s41598-025-88520-4 (PMC11791047; doi:10.1038/s41598-025-88520-4)

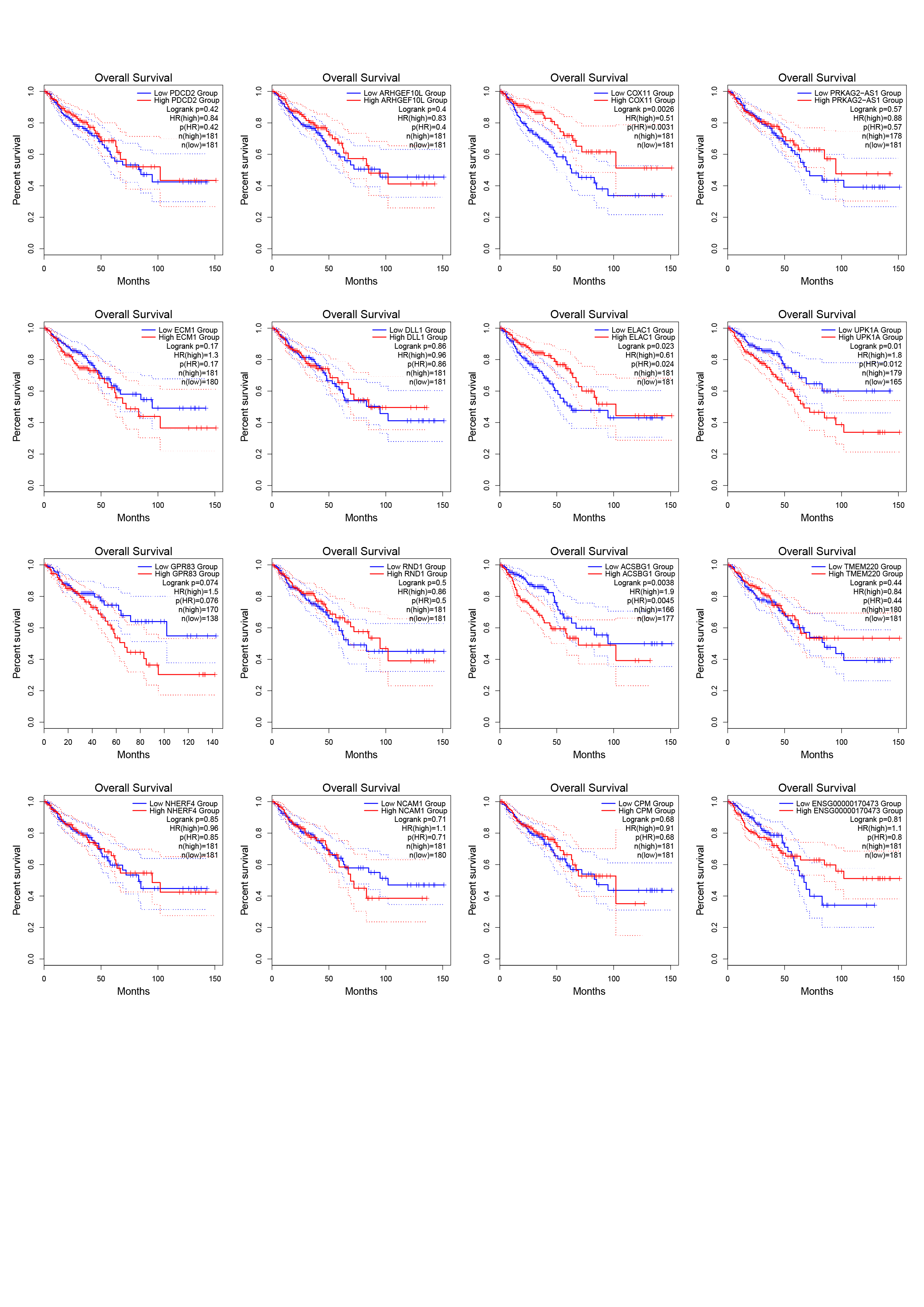

Supplement: Supplementary file 1 — Supplementary Information 1. [file 41598_2025_88520_MOESM1_ESM.png]

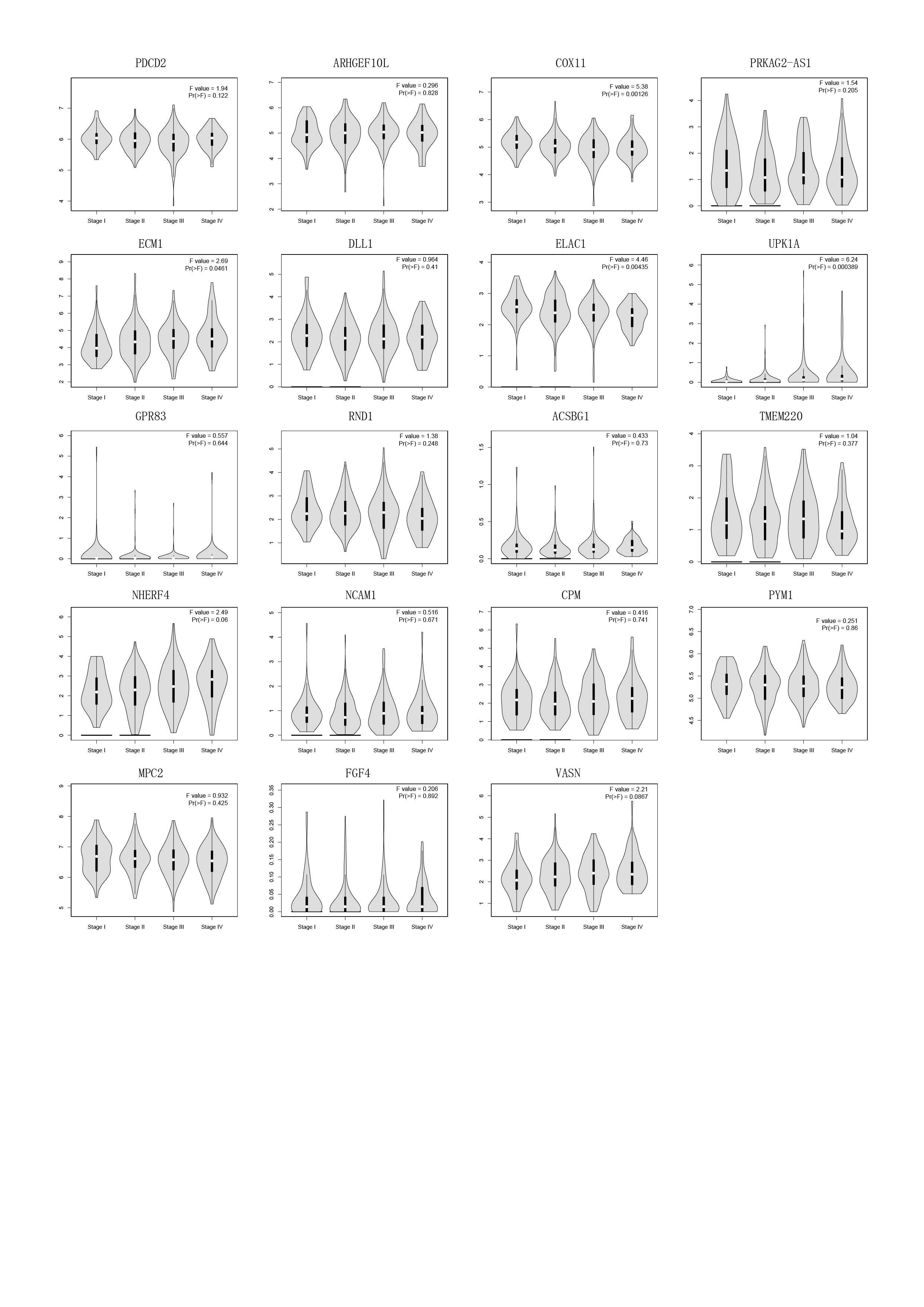

Supplement: Supplementary file 2 — Supplementary Information 2. [file 41598_2025_88520_MOESM2_ESM.png]
