# Supplementary material for: Exploring biomarkers and molecular mechanisms of Type 2 diabetes mellitus promotes colorectal cancer progression based on transcriptomics
Source: Sci Rep. 2025 Feb 3;15:4086. doi: 10.1038/s41598-025-88520-4 (PMC11791047; doi:10.1038/s41598-025-88520-4)
Supplement: Supplementary file 3 — Supplementary Information 3. [file 41598_2025_88520_MOESM3_ESM.docx]

# Supplementary Material

The Supplementary Material for this article can be found online at Submission System.

Supplementary figure 1: KM curves of each gene in the diagnostic model with CRC prognosis.

Supplementary figure 2: Correlation of each gene in the diagnostic model with the pathologic stage of CRC.
